# Protein Folding Activity of the Ribosome (PFAR) –– A Target for Antiprion Compounds 

**DOI:** 10.3390/v6103907

**Published:** 2014-10-23

**Authors:** Debapriya Banerjee, Suparna Sanyal

**Affiliations:** Department of Cell and Molecular Biology, Uppsala University, Box-596, BMC, Uppsala SE-75124, Sweden; E-Mail: debapriya.banerjee@icm.uu.se

**Keywords:** Prion, antiprion compounds, amyloid, protein folding, PFAR, ribosome, ribosomal RNA, domain V, 23S rRNA

## Abstract

Prion diseases are fatal neurodegenerative diseases affecting mammals. Prions are misfolded amyloid aggregates of the prion protein (PrP), which form when the alpha helical, soluble form of PrP converts to an aggregation-prone, beta sheet form. Thus, prions originate as protein folding problems. The discovery of yeast prion(s) and the development of a red-/white-colony based assay facilitated safe and high-throughput screening of antiprion compounds. With this assay three antiprion compounds; 6-aminophenanthridine (6AP), guanabenz acetate (GA), and imiquimod (IQ) have been identified. Biochemical and genetic studies reveal that these compounds target ribosomal RNA (rRNA) and inhibit specifically the protein folding activity of the ribosome (PFAR). The domain V of the 23S/25S/28S rRNA of the large ribosomal subunit constitutes the active site for PFAR. 6AP and GA inhibit PFAR by competition with the protein substrates for the common binding sites on the domain V rRNA. PFAR inhibition by these antiprion compounds opens up new possibilities for understanding prion formation, propagation and the role of the ribosome therein. In this review, we summarize and analyze the correlation between PFAR and prion processes using the antiprion compounds as tools.

## 1. Introduction

Prion diseases are neurodegenerative disorders associated with neuronal cell death and accumulation of the aggregated amyloid form of the prion protein in the brain tissues of mammals [1,2]. These diseases are usually fatal and currently there are no standardized therapeutics available for their treatment [3]. The endogenously expressed prion protein (PrP) can exist in a soluble (PrP^c^) form, which is harmless and involved in neuronal function. Under conditions not fully understood, it changes into the amyloid or Scrapie (PrP^Sc^) form [1]. These two isoforms of the prion protein are structurally distinct. The PrP^c^ is comprised primarily of alpha helices [4], while the PrP^Sc^ form is dominated by beta sheets [5], which pack against each other, resulting in tight amyloid fibrils. The pathology develops when PrP^Sc^ catalyzes the conversion of the normal, soluble PrP^c^ into the PrP^Sc^ amyloid form. The long fibrils cause neuronal death, which are removed by astrocytes, resulting in a sponge-like appearance of the prion-infected brain. The large fibrils break down to smaller fibrils that ‘seed’ further prion propagation [6,7]. Initially, prion formation and propagation were thought to be a protein-only process [2], but newer results suggest the involvement of other agents, mainly nucleic acids [8,9,10,11,12,13,14]. However, the molecular mechanisms of these processes are still poorly understood.

## 2. Antiprion compound screening assays

The lethal nature of prion diseases, coupled with the fact that they are acquired upon exposure to environmental prions through dietary, medical or surgical procedures, makes the search for antiprion compounds difficult. The lack of insight into the molecular mechanism(s) of prion formation is yet another factor which makes screening of compounds with therapeutic potential against the disease challenging. As a consequence, the discovery of compounds with antiprion activity has been rather slow. Antiprion compounds are selected through one or a combination of assays described below. 

### 2.1. Proteinase K assay 

**Figure 1:**
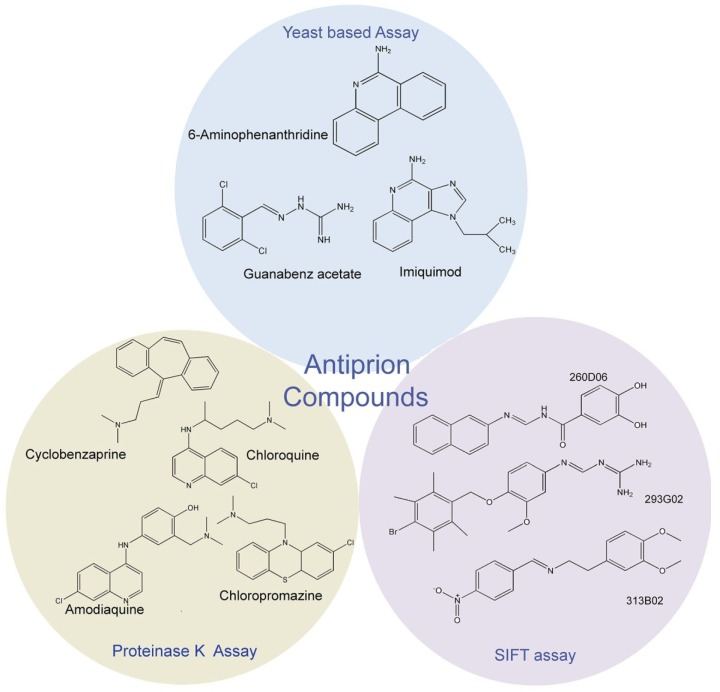
Antiprion compounds identified by different screening assays. Only compounds representing different chemical groups are included here. Antiprion compounds formed by chemical substitutions of these chemical groups are not included, due to space limitations.

The proteinase K assay uses the fact that the aggregated form of most naturally occurring prion proteins is partially resistant to proteinase K digestion [15,16]. In a typical cell-based assay, prion infected cells are treated with test compounds and the lysates are analyzed with proteinase K digestion. Alternatively, in an *in vitro* cell-free assay, the substrate proteins are directly incubated with the test compounds and further treated with proteinase K and then analyzed in SDS-PAGE. The disappearance or retention of the PrP protein band after treatment with proteinase K reveals whether the protein is present in the soluble PrP^c^ or the insoluble PrP^Sc^ form [17], and thus helps to screen compounds which inhibit prion accumulation. This classical assay has been used to screen compound libraries and many compounds with antiprion activity have been identified [17,18]. Some of the representative antiprion compounds identified through the proteinase K assay e.g., chloropromazine and chloroquine, are depicted in Figure 1. However, the technical complexity and the potential health hazard associated with dealing PrP^Sc^ limits the use of the proteinase K digestion assay as a primary and high-throughput assay for screening large libraries of chemical compounds. Another disadvantage is that there are prionogenic systems which are insensitive to proteinase K digestion. 

### 2.2. Fluorescence-Based Assay

This assay is based on a technique known as scanning-for-intensely-fluorescent targets (SIFT). In primary SIFT assay, the formation of a ternary complex between human PrP^Sc^, recombinant mouse PrP (rPrP) labeled with a green fluorescent dye and an L42 monoclonal antibody labeled with a red fluorescent dye is monitored through dual-color confocal microscopy [19]. In the absence of antiprion compounds, molecules of both the L42 antibodies and the rPrP bind to the human PrP^Sc^ aggregates. The resulting complex displays both green and red fluorescence [19]. The L42 monoclonal antibody is specific for human PrP^Sc^; it does not react with mouse rPrP. The presence of an anitprion compound prevents the binding of human PrP^Sc^ to rPrP and the PrP^Sc^ aggregates show a decrease in the green fluorescence with a concomitant shift of fluorescence to the red wavelength. These changes in fluorescence are used as a primary screen for antiprion compounds. The compounds identified in this primary assay are retested in secondary assay involving PrP^Sc^-infected mouse neuroblastoma (N2a) cells instead of purified human PrP^Sc^. Out of eighty compounds identified in the primary (SIFT) assay, only six have been confirmed to be active antiprion agents by the secondary assay. Examples of some compounds identified through the SIFT assay are shown in Figure 1. The SIFT assay allows high-throughput screening of antiprion compounds but can have potential health hazard due to its use of infectious PrP^Sc^ aggregates. The screen also generates false positives in the primary screen. Alternatively, compounds identified in the proteinase K assay, such as quinacrine, are not detected by the SIFT assay.

### 2.3. Yeast-Prion Based Assay 

The technical complexities of the proteinase K assay have been circumvented through the discovery of a safe and fast, yeast-prion based, antiprion compounds screening assay. This yeast-prion based assay uses the protein Sup35, a protein corresponding to a nontoxic [PSI+] prion. In its soluble form, Sup35 participates in translational termination but is unable to do so in its aggregated form. Sup35 plays a role equivalent to the bacterial release factor 3 and thus, translation termination depends on fully functioning soluble Sup35. The aggregation state of Sup35 can be monitored by translation of the *ade1–14* allele that contains an opal stop codon in the open reading frame of ADE1. This gene (*AdeI)* encodes an auxotrophic marker that allows a convenient colorimetric method to monitor the state of the Sup35 protein. When Sup35 is in a soluble state, the ribosome terminates the translation of *ade1–14* allele at the opal stop codon. As a result, adenine production is hampered and the yeast cells do not grow in the adenine deficient medium. This leads to accumulation of metabolic byproducts that produce a red color in rich YPD medium. When Sup35 acquires an insoluble aggregated form, the ribosome often reads through the opal stop codon, resulting in successful adenine production. This enables the cells to grow in adenine deficient medium and moreover, prevents accumulation of metabolic byproducts. As a result, the red color is absent and the colonies appear white in YPD medium. Thus, using conversion of white to red colonies antiprion compounds can be screened in a high-throughput, fast, and cost-effective manner, which is also free from health hazards [20,21]. Besides, because it is based on colorimetric detection, no sophisticated instrumentation is required. 

Compounds identified by Sup35 screening are tested further in another screen based on URE3 prion system, which uses the Ure2 protein [20]. Since Sup35 and Ure2 proteins are distinct from each other in both structure and function, compounds selected through this dual screening system should be active against most yeast strains. The successful antiprion compounds from these two assays are further tested for their ability to clear pathogenic mammalian prion protein in a cell-based assay using MovS6 cells chronically infected with prion. Interestingly, antiprion compounds like quinacrine and chloropromazine, which were originally identified in a proteinase K assay, have been cross confirmed in the yeast-prion based assays [20]. Principal compounds identified in the yeast-prion based assays are depicted in Figure 1. 

## 3. Identification of New Antiprion Compounds in Yeast-Prion Based Assays

**Figure 2:**
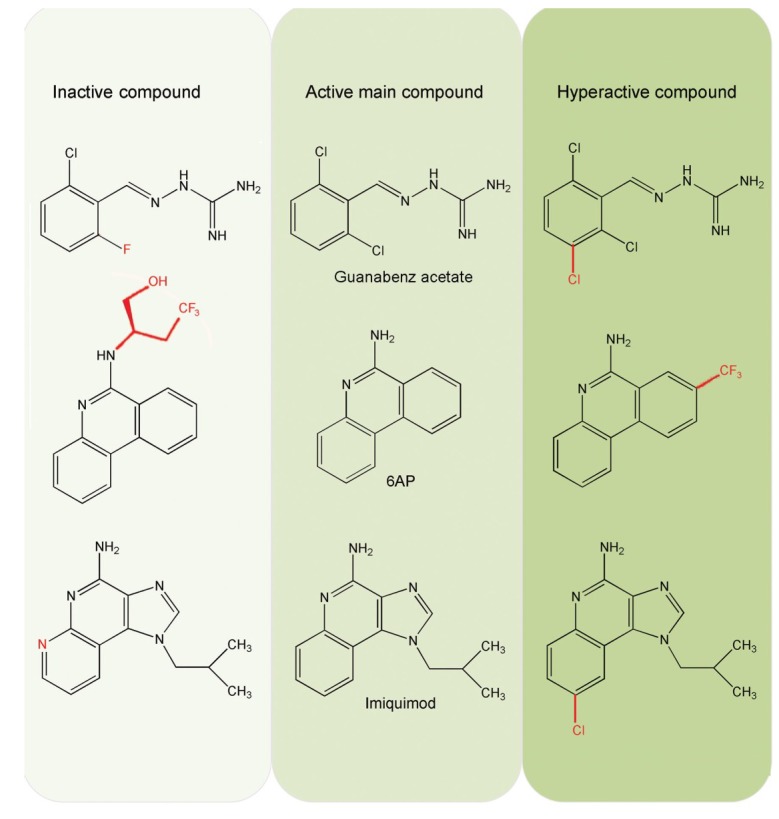
The effect of substitutions on the antiprion activity of 6-aminophenanthridine (6AP), guanabenz acetate and imiquimod derivatives. The primary compounds are in the middle column, while substituted compounds are on the left (inactive) and right (hyperactive). The substitutions are indicated in red.

Huge libraries of compounds have been tested with the yeast-prion based colorimetric assay and several compounds have been identified to be active against prions. Most of them are organic compounds belonging to family kastellpaolitines [20], phenanthridines [20], guanabenzenes [22], and imidazoquinolines [23]. Interestingly, all these compounds, with the exception of GA are aromatic heterocyclic compounds containing nitrogen as the heterocyclic element. This is also true for the compounds identified through other assays (Figure 1). In addition, among the members of the same chemical compound family, antiprion activity is sensitive to substitutions in the parent moiety both with respect to the nature of the substituent group and its position of substitution (Figure 2). 

However, all the derivatives of the primary compound are not equally active; some show increased antiprion activity (hyperactive), while some become completely inactive, reverting to the [PSI+] phenotype in the yeast-prion based assay. The inactive derivatives are named 6APi, GAi *etc.*; the suffix indicates their inactivity in prion assay [24]. The antiprion activity of GA increases upon substitution of an additional chloride group at the 3 position of the benzene ring; this activity is lost when the chlorine atom is replaced with fluorine [22] (Figure 2). Similarly, for the 6AP series, introduction of chlorine and trifluoromethyl group at the 8 position of the phenanthridine ring, (*i.e.*, compounds 6AP8Cl and 6AP8CF_3_, respectively) increases antiprion activity [20]. In contrast, substitution of the amino nitrogen of 6AP with a bulky 2-(butan-1-ol) side chain (compound 6APi) completely abolishes antiprion activity [24] (Figure 2). The antiprion activity of IQ, an imidazoquinoline, is also sensitive to substitution [23]. The introduction of an additional nitrogen atom in the aromatic ring of IQ destroys its antiprion activity, whereas a chloride substitution renders the compound hyperactive (Figure 2).

## 4. Cellular Targets of the Antiprion Compounds 6AP and GA

### 6AP and GA Target the Ribosome by Binding to the Ribosomal RNA (rRNA)

To establish the cellular target of the antiprion compounds 6AP and GA, an affinity chromatography assay (also called “pulled-down assay”) was developed. Affinity matrices were constructed with 6AP and GA chemically linked to the resin via a linker, so that they could physically interact with the target molecules [24,25]. The addition of the linker did not alter much the antiprion activity of the compounds. The open ends of the 6AP-/ GA linkers were then attached to sepharose beads. Control matrices were prepared with sepharose beads quenched in ethanol amine. Crude extracts of the [PSI+] budding yeast, porcine brain or PrP infected MovS6 cells were incubated with the 6AP-/GA-/control beads so that the target molecules can bind to it [24,25]. These beads were then packed into columns and the nonspecific binders were removed by extensive washing. Then, the specific binders were eluted by adding free 6AP/GA to the column, which competitively removed the specific binders from the 6AP-/GA beads. The eluted samples were analyzed by SDS-PAGE followed by Western blotting using antibodies against Sup35p (the protein corresponding to the [PSI+] prion), Ure2 (the protein corresponding to [URE3] prion) or PrP. Surprisingly, although the prion proteins were clearly detectable in crude cell extracts, they could not be detected among the components eluted from the 6AP-/GA- beads [24,25]. This suggests that there is no direct interaction between 6AP/GA and the prion proteins. In addition, using highly sensitive Protein Misfolding Cyclic Amplification (PMCA) assay it was shown that 6AP and GA do not affect the *in vitro* conversion of PrP^c^ to PrP^Sc^ [24]. This is further evidence that there is no direct interaction between prion proteins and 6AP or GA. 

The results of the pulled-down assay led to another important observation. The Western blot did not show bands corresponding to the prion proteins, but did reveal bands corresponding to several ribosomal proteins. This would be possible only if 6AP and GA would target rRNA and consequently pull-down the whole ribosome from the 6AP-/GA beads. To test that crude extracts of [PSI+] yeast were treated with RNase and then affinity chromatography was conducted as described. As expected, no bands corresponding to the ribosomal proteins could be seen in the Western blot. This result confirmed that the binding of ribosomal proteins to the 6AP/GA is rRNA-dependent and ribosome is the cellular target of 6AP and GA. Thus, 6AP and GA inhibit prion formation in an indirect pathway.

## 5. 6AP, GA and IQ Inhibit Protein Folding Activity of the Ribosome (PFAR)

### 5.1. PFAR

**Figure 3:**
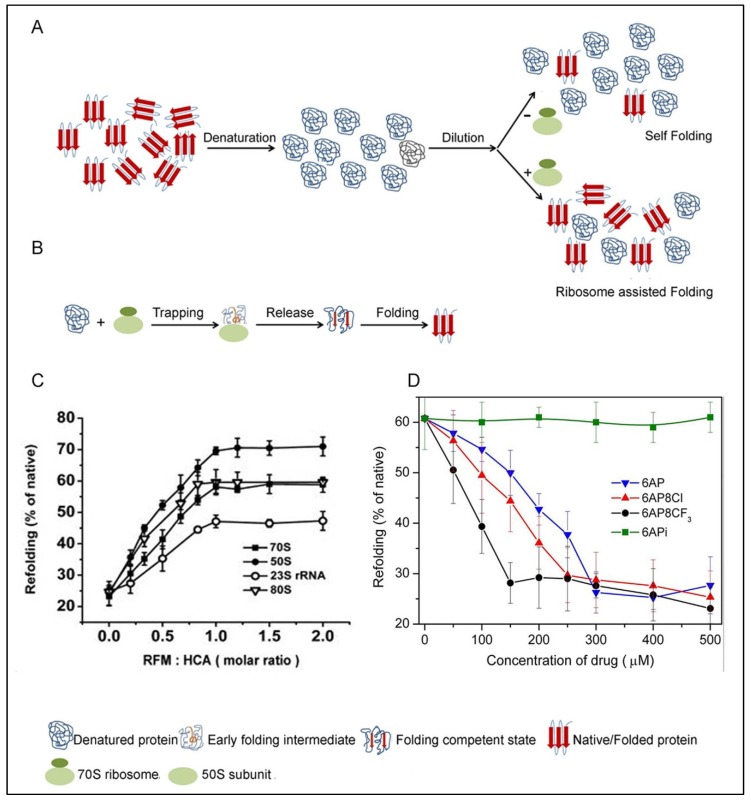
(**A**) Scheme of PFAR (Protein Folding Activity of the Ribosome) assay; self-folding and ribosome assisted folding; (**B**) Two step trapping and release model of PFAR; (**C**) Refolding of HCA with different RFMs and at various RFM: HCA ratios (Reproduced with permission from [44]. Copyright © 2011 Elsevier Masson SAS. All rights reserved), (D) Inhibition of PFAR by 6AP derivatives using HCA as the model protein. (Reproduced with permission from [45]. Copyright © 2014 Elsevier Masson SAS. All rights reserved).

In addition to its classical role in protein synthesis, the ribosome also acts as a protein folding modulator (for review see [26,27,28]). The protein folding activity of the ribosome (PFAR) was first demonstrated by DasGupta *et al.* who found that denatured proteins could be refolded to their functionally active form by including ribosomes in the refolding buffer [26,29,30] (Figure 3A). Ribosome-assisted folding has been observed with many classes and sources of proteins [31,32,33,34,35] and ribosomes from all three kingdoms of life have shown to possess protein folding activity [31,32]. Further studies reveal that PFAR is a function of the large ribosomal subunit [34,35,36]. The active site of PFAR is located at the subunit interface of the large subunit composed of domain V of the largest rRNA (23S in bacteria, 25S/28S in eukaryotes) [33,36]. It is interesting to note that the domain V rRNA fragment alone can assist folding of denatured proteins even when transcribed *in vitro* [33,37,38,39]. The domain V rRNA also constitutes the peptidyl transferase center (PTC), where peptide bonds are formed on the ribosome. The sequence and the secondary structure of the domain V rRNA are highly conserved; in particular, the central loop region is very similar in ribosomes from all sources [40]. Thus, the fact that the domain V rRNA hosts the active site for PFAR has evolutionary significance. 

Based on the studies with *in vivo* and *in vitro* protein folding using bacterial ribosomes, it appears that PFAR occurs in a stepwise manner. Initially, the full-length polypeptide chain splits a non-translating ribosome into subunits [41,42] and harbors itself on the large central loop of the domain V rRNA to attain a folding competent state [33,38]. The folding competent protein is then released from the ribosome with assistance from other parts of the domain V rRNA to the cytosol where it assumes its final active conformation [33,38] (Figure 3B). Chaperones like DnaK and trigger factor, which are generally associated with the ribosome, might also assist protein folding at this stage [43]. 

### 5.2. The PFAR Assay

In a typical PFAR assay, the denatured protein of choice is refolded by dilution of the denaturant (6M guanidine hydrochloride or 8M urea), in a suitable buffer, in the absence (self-folding) or presence of ribosomal folding active components or ribosomal folding modulators (RFM), (70S ribosome/50S ribosomal subunit/23S rRNA/*in vitro* transcribed domain V rRNA/the eukaryotic homologs) (Figure 3A) [24]. The folding is followed by measuring the enzymatic activity of the substrate protein in standard colorimetric assays using a spectrophotometer or multimode plate reader. The extent of folding is estimated by comparison with the activity of the native protein stored undiluted at 0°C, which is considered as 100%. One frequently used model protein in the PFAR assay is human carbonic anhydrase (HCA). Other enzymes such as lactate dehydrogenase, malate dehydrogenase, lysozyme *etc.* have also been used as substrate proteins and a list can be found in the review by Das *et al.* [26]. 

The folding yield varies with the RFMs. Highest refolding, irrespective of the model protein, is achieved with 50S subunit as RFM, followed by 70S ribosome, 23S rRNA, and domain V rRNA transcribed *in vitro* [32,35,44] (Figure 3C). The eukaryotic RFMs also show the same order of activity in assisting protein folding. Interestingly, maximum refolding is achieved when the substrate protein and the RFMs are present in a 1:1 ratio (Figure 3C) [44]. Further increase in amount of RFM does not increase the amount of folded protein. Also, the RFMs do not affect the rate of folding; instead they only modulate a larger fraction of substrate proteins to achieve native state compared to self-folding. 

### 5.3. Inhibition of PFAR by 6AP, GA and IQ and Their Derivatives

The antiprion compounds 6AP, GA and IQ do not affect self-folding of the proteins. This is consistent with the fact that they do not interact directly with the proteins. In contrast, they strongly inhibit PFAR in a concentration dependent manner; the concentration of antiprion compound required for complete inhibition of PFAR varies according to the efficiency of the compounds in PFAR inhibition [23,24,44]. When PFAR is completely inhibited by 6AP/GA/IQ the amount of refolded protein matches with the self-folding. A comparison of the inhibitory activity of the three compounds shows that IQ is a better inhibitor of PFAR than 6AP and GA.

The effect of different derivatives of 6AP, GA and IQ on PFAR has also been studied [23,24,44,45]. For all three compounds, the derivatives inactive in yeast-prion based assay (e.g., 6APi) (Figure 2) are also unable to inhibit PFAR [45]. Not only that, the 6AP derivatives inhibit PFAR in the same order as their antiprion activity, *i.e.*, 6AP8CF_3_ > 6AP8Cl > 6AP > 6APi (no inhibition) [45]. The stronger the antiprion action of a compound, the greater is its ability to inhibit PFAR (Figure 3D). This suggests that there is a strong correlation between antiprion activity and PFAR. 

## 6. Mode of Inhibition of PFAR by 6AP and Its Derivatives

### 6.1. 6AP Inhibits PFAR by Binding to Specific Sites on the Domain V rRNA

**Figure 4:**
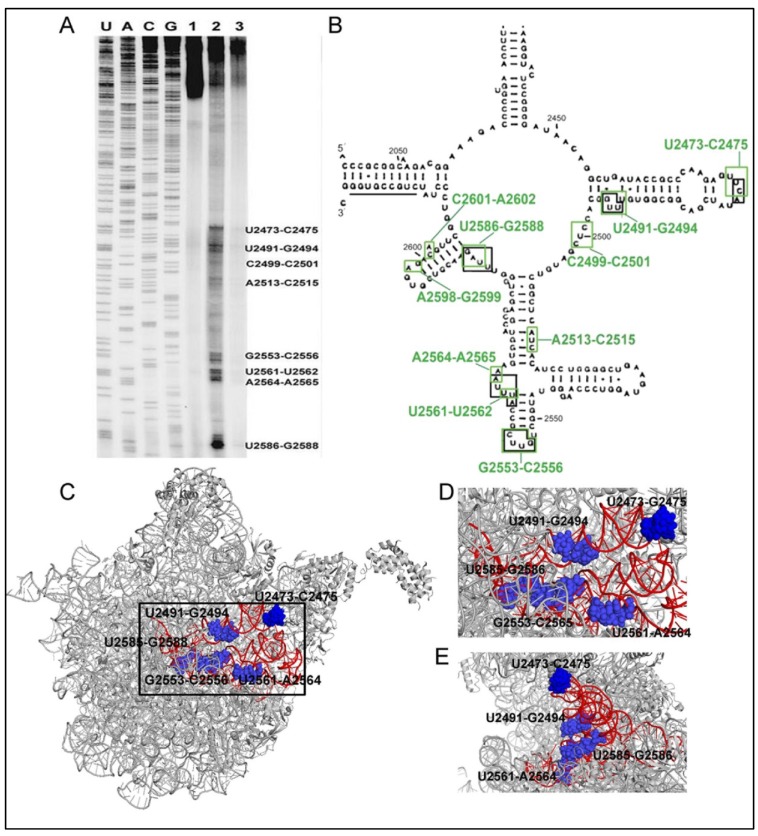
(**A**) Sequencing gel showing the binding (road block) sites of 6AP (lane 2) and 6APi (lane 3) on the domain V rRNA identified by primer extension after UV-crosslinking sites. Lane 1 is the control without 6AP/6APi. (**B**) Binding sites of 6AP and the protein folding substrates in domain V rRNA. The green and the black boxes indicate 6AP and the protein binding sites, respectively. (**A**–**B**), The research was originally published in JBC [46]; Reproduced with permission. © The American Society for Biochemistry and Molecular Biology); (**C**) The position of the binding sites in the 50S subunit of *E. coli* ribosome. (PDB ID: 3UOS) The red highlighted portion represents residues 2435–2668 of domain V rRNA where most of the binding sites (marked with blue spheres and labeled with residue numbers) are located; (**D**) and (**E**) The enlarged view of the binding pocket (black box in **C**) from front (**D**) and side (**E**) view.

The pulled-down assay showed that the antiprion compounds (6AP and GA) do not interact with the prion protein [24] but bind to the ribosome and particularly rRNA. Since PFAR has been localized on the domain V rRNA of the ribosomal subunit and 6AP inhibits PFAR, one would naturally expect that 6AP binds to the same domain of the rRNA. The exact binding sites of 6AP to domain V rRNA was determined through UV cross-linking followed by primer extension assay [46]. In this experiment, 6AP was cross-linked to the *in vitro* transcribed domain V rRNA by UV irradiation. Then using domain V specific primers, primer extension was performed with reverse transcriptase on this 6AP-domain V rRNA complex. The primer extension was stopped at the 6AP cross-linked sites, which were identified as distinct bands (also called ‘road blocks’) on the sequencing gel (Figure 4A) [46]. The major 6AP binding sites are U2473-C2475, U2491-G2494, G2553-C2556, U2561-A2564, U2585-G2586, marked with green boxes on the secondary structure of domain V rRNA in Figure 4B. Interestingly, in a parallel experiment, 6APi produced only nonspecific bands suggesting that it does not bind to the domain V rRNA [46]. 

### 6.2. 6AP Binding Sites Overlap with Protein Binding Sites

Primer extension studies were also done to identify the binding sites of the protein substrates on the domain V rRNA. For that, three unrelated substrate proteins (human carbonic anhydrase, bovine carbonic anhydrase and dihydrofolate reductase), freshly diluted from denaturation mix, were UV cross-linked to the domain V rRNA [46]. Interestingly, all three proteins show a common binding map on domain V rRNA. Furthermore, a comparison of the protein binding maps with a 6AP interaction map on domain V rRNA shows a major overlap between the two (Figure 4B). These sites, marked with black boxes in Figure 4B, are U2473–C2475 U2491–G2494, G2553–C2556, U2561–A2564, U2585–G2586. When viewed on the whole ribosome, these sites appear as a well-formed pocket at the subunit interface (Figure 4C–E). These sites are largely exposed in the free 50S. The sites U2473–C2475 and U2561–A2564 are completely exposed at the subunit interface, and thus clearly visible in the side view of the 50S subunit (Figure 4D–E). The strong overlap between 6AP and protein binding sites suggest competitive interaction between the two. Since 6AP is a small molecule, it is likely that several 6AP molecules bind simultaneously onto the domain V rRNA and thereby obstruct the access of the protein substrates to the PFAR active sites. The competitive inhibition of PFAR has been further confirmed by mutagenesis and kinetic assays.

### 6.3. Both PFAR and 6AP/Protein Substrate-Binding are Sensitive to Mutations on Domain V rRNA

To study the effect of specific nucleotides on PFAR, mutations were introduced at the common 6AP- and protein binding sites on the domain V rRNA [46]. Both the bacterial (*E. coli* 23S rRNA) and eukaryotic (*Saccharomyces cerevisae* 25S rRNA) domain V rRNA were subjected to mutagenesis on the homologous sites. For comparison, mutations were also introduced in some random sites on domain V rRNA. All mutated domain V rRNAs were tested for their protein folding activity in PFAR assay. In parallel, some of the mutated domain V rRNAs were UV cross-linked with 6AP and human carbonic anhydrase (HCA) as a protein substrate (in separate reactions) and were checked by primer-extension. 

Most of the altered domain V rRNAs with mutation in 6AP- or protein binding sites were defective in PFAR [46]. The biggest defect was observed for the mutations (individual or multiple) at positions 2492–2494, 2561–2562, and 2586–2588 in the *E. coli* domain V rRNA. Corresponding mutations in the eukaryotic domain V rRNA also showed highly defective protein folding. Control mutations at residues 2486–2488 on 23S rRNA and equivalent positions on 25S rRNA, which did not correspond to protein or 6AP binding sites, showed no defect in PFAR. 

When the mutant domain V rRNAs defective in PFAR were tested by UV-crosslinking followed by primer extension analysis, distinct changes in the binding maps of both HCA and 6AP were noticed [46]. For the mutant UUG2492–2494CCA, the bands corresponding to this site disappeared for both HCA and 6AP. For the other mutant, UAG2586–2588CCA, 6AP binding bands were totally absent and HCA showed much weaker bands. Thus, it can be inferred that PFAR depends on interaction with specific residues on domain V of 23S/25S rRNA, which are also the binding sites for 6AP. 6AP blocks those sites and thereby prevents the protein substrates from binding to the PFAR-specific residues on the domain V rRNA.

### 6.4. Competitive Inhibition of PFAR by 6AP and GA

As shown in Figure 4, the 6AP binding map overlaps the protein binding map, which indicates that 6AP and the protein substrates for PFAR compete for the same sites on domain V rRNA [46]. To test this hypothesis, the mode of inhibition of PFAR by 6AP and GA has been studied by conventional biochemistry. The PFAR assay was conducted with 70S/80S ribosome or domain V rRNA as the RFMs and HCA as a substrate, in the presence of these two antiprion compounds [44,46]. The yield of the folded protein as well as the time course of refolding was monitored. Irrespective of the RFM, increasing concentration of 6AP and GA gradually decreases the yield of RFM-assisted refolding to the level of self-folding, but the rate of refolding (measured by enzymatic activity assay) remains more or less unaltered. This situation can be compared with the typical competitive inhibition where in the presence of the inhibitor the constant for substrate binding (*K_M_*) increases, but the maximal rate of the reaction (*k_cat_*) remains constant. Furthermore, the inhibition of PFAR by 6AP and GA can be reversed by addition of RFMs in excess. These data confirm that 6AP and GA inhibit PFAR in a competitive manner. 6AP and GA occupy the binding sites on domain V rRNA, which also constitute the active site for PFAR. When the active sites are blocked with 6AP/GA, the protein substrates fail to bind to domain V rRNA and fold in self-folding pathway. Since 6AP/GA do not interact with the protein substrate, they do not affect self-folding of the proteins. It is likely that the domain V rRNA and protein substrate interactions are transient and the protein actually folds to the active state after being released from the RFMs. This explains why the kinetics of refolding of HCA, monitored by measuring its enzymatic activity, is not affected by the presence of RFMs or the PFAR inhibiting antiprion compounds [44,46].

### 6.5. Comparison of 6AP Derivatives for Their Affinity to Domain V rRNA

**Figure 5:**
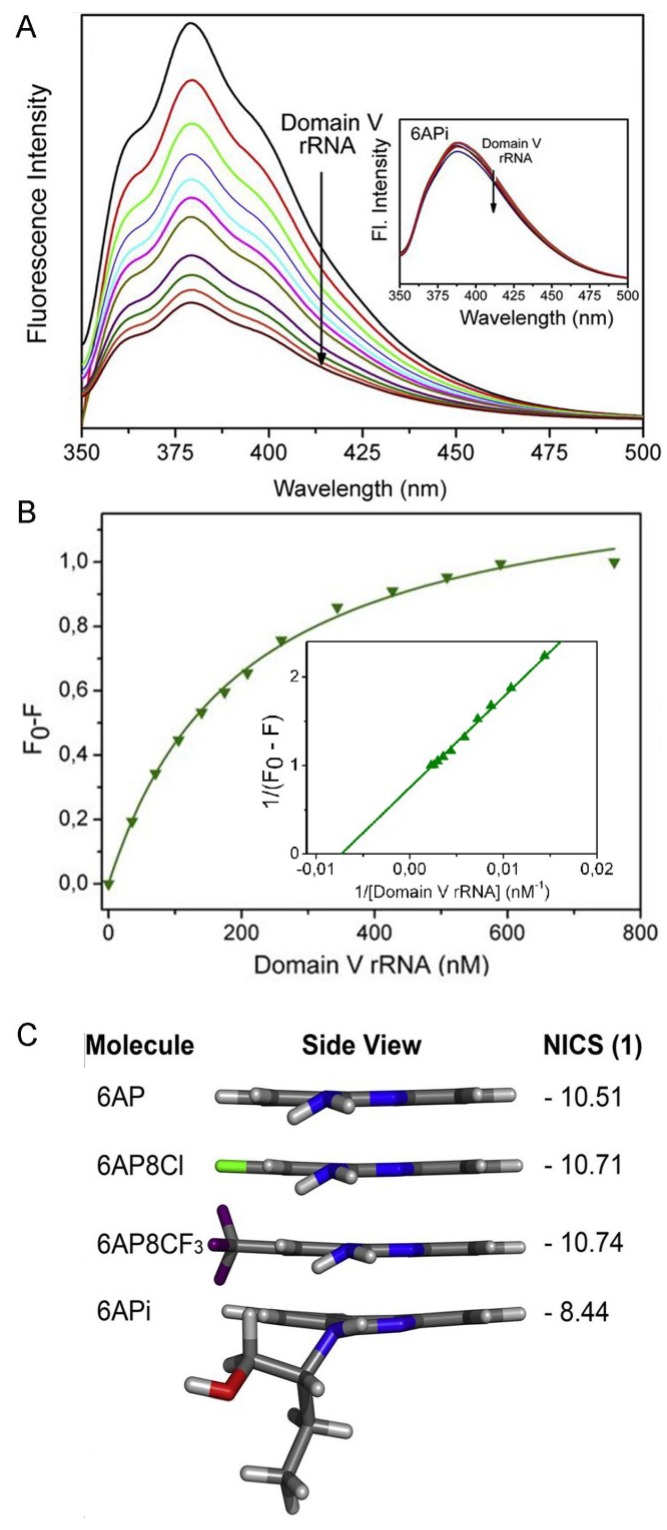
(**A**) Quenching of 6AP fluorescence in the presence of domain V rRNA. Inset shows 6APi fluorescence, which does not quench by addition of domain V rRNA; (**B**) Saturation binding curve of 6AP with domain V rRNA. The double reciprocal plot of the binding curve is in the inset, the K_D_ value is determined from the reciprocal of the X-intercept; (**C**) Lateral view of the 6AP derivatives and corresponding values of Nucleus independent chemical shift (NICS). The figure is reproduced with permission from [45]*.* Copyright © 2014 Elsevier Masson SAS. All rights reserved.

In parallel with the UV cross-linking studies, the intrinsic fluorescence of 6AP derivatives has been used to study their binding to domain V rRNA [45]. This is the first study using the fluorescence property of 6AP in such manner and it was found that the intrinsic fluorescence of 6AP, 6AP8Cl and 6AP8CF_3_ decreases gradually upon addition of domain V ribosomal RNA (Figure 5A). Fluorescence quenching has been used to study molecular interaction in many systems. The quenching of 6AP/6AP8Cl/6AP8CF_3_ fluorescence with domain V rRNA suggests a direct interaction between the 6AP derivatives and the domain V rRNA. In contrast, no change in fluorescence was observed with 6APi, suggesting that it does not interact with the RNA at all (Figure 5A, inset). The saturation binding curves of 6AP, 6AP8Cl and 6AP8CF_3_ are derived from the change in fluorescence (Figure 5B). The equilibrium binding constants or *K_d_* values estimated for 6AP8CF_3_, 6AP8Cl and 6AP are 10 ± 0.25 nM, 100 ± 4.7 nM and 140 ± 7.2 nM, respectively [45], which indicates that 6AP8CF_3_ is the strongest binder of domain V rRNA, followed by 6AP8Cl and 6AP. Interestingly, the 6AP derivatives show the same order of binding affinity to domain V rRNA, *i.e.*, 6AP8CF_3_ > 6AP8Cl > 6AP > 6APi (no binding), as seen for the antiprion activity of the compounds and also for PFAR inhibition. This reconfirms that 6AP and its derivatives inhibit PFAR by binding to domain V rRNA and that PFAR is implicated in prion formation or propagation. 

### 6.6. Density Functional Theory (DFT) Calculations Explain why 6APi Cannot Bind to Domain V rRNA

Density functional theory (DFT) calculations have been used to explain the differential binding behavior of 6AP and its derivatives towards domain V rRNA [45]. Nucleus independent chemical shift (NICS) values, which reflect aromaticity of the compounds that is indirectly related to their planarity, have been estimated for the 6AP derivatives. 

More negative the NICS value, higher is the planarity of the compound. Also, more planar the compounds, the better binders they are to nucleic acids. The calculated NICS values for 6AP, 6AP8Cl, 6AP8CF_3_ and 6APi are listed in Figure 5C, which shows that the attachment of chloro (Cl) and trifluoromethyl (CF_3_) groups at the 8 position in 6AP8Cl and 6AP8CF_3_ does not affect the planarity of the phenanthridine ring. In fact, these substitutions enhance the aromatic ring current and hence, these two 6AP derivatives are better binders of domain V rRNA than 6AP. However, substitution of the amine nitrogen of 6AP with a 2-(butan-1-ol) group makes 6APi nonplanar. Moreover, the bulky substitution extends from the plane of the phenanthridine ring, which could create steric hindrance for binding. Thus, the deviation of planarity in 6APi and the steric hindrance due to the bulky substitution inhibits 6APi from binding to domain V rRNA (Figure 5C). As a result, 6APi cannot inhibit protein substrates from binding to domain V rRNA and thus cannot inhibit PFAR. The incapability of 6APi in domain V rRNA binding and PFAR inhibition explains its inactivity as antiprion compound.

## 7. PFAR and Prion Correlation: Conclusions and Future Perspectives

**Figure 6:**
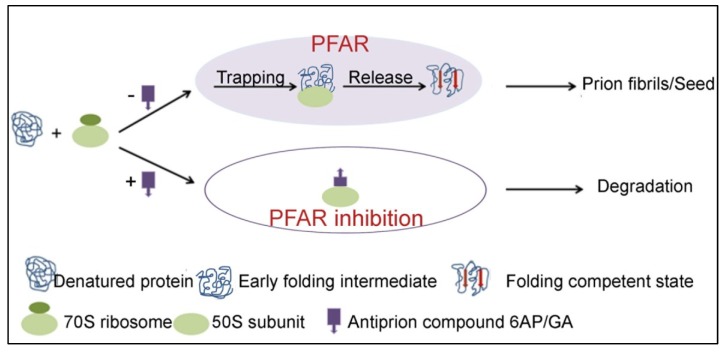
The current model describing involvement of PFAR in prion processes and the role of the antiprion compounds (6AP/GA/other compounds with similar activity) in it.

The inhibition of PFAR by the antiprion compounds 6AP, GA, and IQ suggests that the ribosome and particularly, PFAR might have a role in prion formation and/or propagation. Although the molecular mechanisms are not yet available, our understanding of PFAR and its involvement in prion propagation have advanced through the recent investigations. We now know that PFAR is dependent on the domain V rRNA, which is a highly conserved domain in all kingdoms of life. The ribosome assists an unfolded protein to fold correctly by trapping it to specific sites on the domain V of rRNA and then releasing it in a folding competent state. For prion proteins, there can be at least two models to explain how PFAR can influence the prion processes. PFAR could be involved in conversion of the prion proteins to the amyloid forms or its precursors. Alternatively, PFAR could be involved in breaking long prion fibrils into smaller prion seeds; thereby facilitating prion propagation. Experiments designed to test these two models will establish the true correlation between PFAR and prion diseases. Recent experiments also allowed us to understand how 6AP and GA (and their active antiprion derivatives) work in blocking prion processes. These antiprion compounds are primarily rRNA dependent inhibitors of PFAR. When these compounds inhibit PFAR by binding to domain V rRNA, the prion proteins probably fold into the unstructured aggregates and get degraded by cellular proteases, thereby blocking prion formation/propagation. The current view of PFAR and its role in prion processes is illustrated in Figure 6. We also demonstrate the interplay between 6AP/GA/other antiprion compounds (working with the same principle) and PFAR to explain their activity as antiprion compounds.
